# Cover

**DOI:** 10.4269/ajtmh.1103_Supplcover

**Published:** 2024-03-05

## Abstract

Top: Sam Agbor Enow (left) supervises the preparation of malaria medicine injectable artesunate by Nurse Haoua Abba at Bogo District Hospital, Far North Region, Cameroon, September 23, 2021. Sam has been trained to provide Outreach Training Supportive Supervision Plus (OTSS+) to ensure that healthcare providers know, and can follow, national guidelines for correctly diagnosing and treating malaria. Photo Credit: Mwangi Kirubi, PMI Impact Malaria.

Bottom left: Nurse Gertrude Doku teaches her team how to conduct a malaria rapid–diagnostic test (mRDT) at the Tinkong Community–Based Health Planning and Services (CHPS) Compound in Ghana. Photo Credit: Emmanuel Attramah, PMI Impact Malaria.

Bottom right: A health provider in Zambia provides care to a child with malaria while an Outreach, Training, and Supportive Supervision (OTSS) visit takes place. OTSS is a quality assurance approach at the health–facility level, that uses standard automated checklists centered on continuous improvement of the competencies of health providers in malaria diagnosis and treatment. Photo Credit: PMI Impact Malaria

